# Missing Nurse Bees—Early Transcriptomic Switch From Nurse Bee to Forager Induced by Sublethal Imidacloprid

**DOI:** 10.3389/fgene.2021.665927

**Published:** 2021-06-17

**Authors:** Yun-Ru Chen, David T. W. Tzeng, Chieh Ting, Pei-Shou Hsu, Tzu-Hsien Wu, Silin Zhong, En-Cheng Yang

**Affiliations:** ^1^Department of Entomology, National Taiwan University, Taipei, Taiwan; ^2^School of Life Sciences, The Chinese University of Hong Kong, Hong Kong, China; ^3^Miaoli District Agricultural Research and Extension Station, Council of Agriculture, Executive Yuan, Gongguan, Taiwan

**Keywords:** honey bee, imidacloprid, sublethal dosage, transcriptome, precocious foraging

## Abstract

The environmental residue/sublethal doses of neonicotinoid insecticides are believed to generate a negative impact on pollinators, including honey bees. Here we report our recent investigation on how imidacloprid, one of the major neonicotinoids, affects worker bees by profiling the transcriptomes of various ages of bees exposed to different doses of imidacloprid during the larval stage. The results show that imidacloprid treatments during the larval stage severely altered the gene expression profiles and may induce precocious foraging. Differential expression of foraging regulators was found in 14-day-old treated adults. A high transcriptome similarity between larvae-treated 14-day-old adults and 20-day-old controls was also observed, and the similarity was positively correlated with the dose of imidacloprid. One parts per billion (ppb) of imidacloprid was sufficient to generate a long-term impact on the bee’s gene expression as severe as with 50 ppb imidacloprid. The disappearance of nurse bees may be driven not only by the hive member constitution but also by the neonicotinoid-induced precocious foraging behavior.

## Introduction

With high pollinating efficiency, workers of the honey bee, *Apis mellifera*, are the most important pollinators for agricultural crops as well as wild plants (Calderone, 2012; van der Zee et al., 2012; Garibaldi et al., 2013, 2016; Hung et al., 2018). Although honey bees may be exposed to different types of agricultural chemicals, previous studies evaluating the impacts of pesticides found that a group of insecticides called neonicotinoids may be particularly hazardous to honey bees (Goulson, 2013; Lu et al., 2014; Sandrock et al., 2014; Goñalongs and Farina, 2015). While foragers are more frequently in contact with the pesticides, the whole colony is also under the threat of sublethal neonicotinoids as neonicotinoid-contaminated nectar and pollen are delivered to the colony through foraging activities (Chauzat et al., 2006; Škerl et al., 2009; Codling et al., 2016; Mitchell et al., 2017; Böhme et al., 2018).

A sublethal dose of imidacloprid, one of the major neonicotinoids mostly used around the world, may not cause acute bee death, but it can generate chronic irreversible damage to the bee colony. Chronic exposure to sublethal doses of imidacloprid can generate abnormal behavior or physiological disorders in bees (Goulson, 2013; Lu et al., 2014; Sandrock et al., 2014; Goñalongs and Farina, 2015). A long-term investigation revealed that honey bee colony health and overwintering success are negatively affected by exposure to field doses [20–100 parts per billion (ppb)] of imidacloprid and that colony survival is correlated with the amount of imidacloprid (Dively et al., 2015; Woodcock et al., 2017; Wood et al., 2018).

For foragers, exposure to sublethal imidacloprid doses shortens their lifespan, permanently impairs their olfactory-associated leaning ability, and alters both their foraging behavior as well as foraging frequency (Decourtye et al., 2003, 2004; Faucon et al., 2005; Yang et al., 2008; Schneider et al., 2012; Zhang and Nieh, 2015). A higher rate of failure in homing results in a reduced number of foragers. To compensate for the shortage of foragers, nurse bees develop and reach an early behavioral maturation to generate young, precocious foragers (Beshers and Fewell, 2001; Leoncini et al., 2004; Sanchez-Bayo and Goka, 2014; Perry et al., 2015). Under these stressful conditions, the number of worker bees gradually decreases, resulting in colony collapse (Sanchez-Bayo and Goka, 2014; Perry et al., 2015; Ushitani et al., 2016).

Larvae and nurse bees are affected by consuming neonicotinoid-contaminated pollen or nectar. Honey bee larvae can tolerate higher doses of imidacloprid than nurse bees [LD_50_ = 4.17 μg, LC_50_ = 138.84 parts per million (ppm)] (Dai et al., 2017), but larval synapse development is affected by low doses. After exposure to 10 ppb imidacloprid, honey bee larvae pupation and eclosion rates are not affected. However, the developed adults show a reduced synaptic density in the brain region responsible for olfactory and visual function, and the olfaction-related learning behavior is permanently impaired (Yang et al., 2012; Peng and Yang, 2016). With continuous exposure, all these physiological and behavioral disorders induced by the sublethal dose of neonicotinoids can be sustained throughout the lifespan of workers and exhibit in different syndromes with the progress of bee development.

Due to difficulties in observation, only a few studies report the impact of imidacloprid on nurse bee behavior; they report that nurse bees show less activity and social interaction after being exposed to a low dose of imidacloprid (Medrzycki et al., 2003; Forfert and Moritz, 2017). From the molecular perspective, genes with functions related to metabolism, biosynthesis, and royal jelly synthesis are affected after exposure to sublethal doses of imidacloprid or other neonicotinoids (Aufauvre et al., 2014; Shi et al., 2017a,b; Wu et al., 2017; Christen et al., 2018; Wang et al., 2018). Most molecular studies suggest an immediate response after treatment; nevertheless, it is unclear whether the impact is long-lasting. In this report, we comprehensively evaluate the short- and long-term effects of sublethal doses of imidacloprid on honey bees of different ages following exposure during the larval stage. We used next-generation sequencing (NGS) to investigate the transcriptome of 9-day-old larvae and 0-, 7-, 14-, and 20-day-old adults. Different concentrations of imidacloprid solutions, including 1, 10, and 50 ppb, were applied to confirm the dosage effect and determine the lowest effective concentration of imidacloprid that could alter honey bee gene expression.

## Materials and Methods

### Honey Bees

Honey bees were purchased from a local bee keeper at Hsinchu area and maintained at National Taiwan University and Miaoli District Agricultural Research and Extension Station (Council of Agriculture, Executive Yuan). Colonies contained at least eight honeycomb frames, a normal egg-laying queen, larvae, and pupae, as well as pollen and honey. Bee stability was continuously monitored. Pollen cake (45% sucrose, 30% pollens, 15% soybean powder, and 10% H_2_O) and 50% sucrose solution were provided during the non-flowering season. The area nearby the colonies was confirmed as not using neonicotinoid pesticides.

### Imidacloprid Solution Preparation

Imidacloprid powder (Sigma) was dissolved in 100% DMSO to a final concentration of 100 ppm and then diluted to 1, 10, and 50 ppm with DMSO. All solutions were aliquoted in 1.5-ml tubes and stored at −20°C until further use. Imidacloprid working solutions were freshly prepared every day before feeding. To prepare working solutions, 1, 10, and 50 ppm imidacloprid stock solutions were diluted with ddH_2_O to final concentrations of 1, 10, and 50 ppb (final concentration of DMSO is 0.1%).

### Imidacloprid Feeding Assay

The imidacloprid feeding assay and sample collection were performed during October to December 2016. For sample collection, two colonies were selected. For each colony, at least 300 larvae were used. The queen was restricted in one frame to lay eggs for 16 h to synchronize the age of experimental individuals. After the larvae hatched (defined as 1-day-old larvae), 1 μl of 1, 10, or 50 ppb of imidacloprid solution was added to each cell. This feeding procedure was performed daily for 4 days, from 1- to 4-day-old larvae, to mimic the number of days honey bee larvae consume bee bread. For each experimental group, a total of 4 μl of imidacloprid solution was applied into every cell, resulting in final amounts of imidacloprid consumed per larva of 0.004, 0.04, and 0.2 ng, respectively. Parallel controls were larvae fed with either ddH_2_O or 0.1% DMSO (see Supplementary Figure 1A for the experiment procedure). At 10 days after the cells were capped, pupae were pulled out and kept separately according to the treatments in 96-well plates at 37°C until eclosion. Bees from two different beehives were pooled as one group for the labeling and sampling procedure. Newly emerged adults were labeled on the thorax with different colors of acrylic paint based on the treatment and then randomly released into two beehives. Bees from different colonies were mixed and randomly assigned for the following experiment. The results represented in this report were the integrated effects from two hives of bee. Sampling was performed on 9-day-old larvae and 0- (newly emerged), 7-, 14-, and 20-day-old adults. The 0-, 7-, 14-, and 20-day-old adults conduct the general tasks of cell cleaning, brood rearing, pollen/nectar processing, and foraging, respectively (Seeley, 1982). For each age of worker bee, three biological replicates were collected, five individuals per replicate to final 15 individuals per age per treatment. Samples were stored in RNAlater (Invitrogen, Carlsbad, CA, United States) at −20°C for subsequent RNA extraction.

### RNA Extraction and RNA-seq Library Preparation

RNA from whole larvae and adults’ heads was extracted using an RNeasy mini kit (Qiagen, Hilden, Germany) following the user manual. RNA quality was checked in an agarose gel, with no significant degradation of *28S* and *18S* bands. RNA quantity was measured using the NanoDrop 2000 UV-Vis spectrophotometer (Thermo Fisher Scientific, Waltham, MA, United States). For each sample, at least 10 μg of total RNA was used for RNA-seq library construction. The library construction was performed according to Zhong et al. (2011). Briefly, polyA RNA enrichment was performed using Oligo d(T)25 Magnetic Beads (NEB, Ipswich, MA, United States), then eluted and fragmented simultaneously in 2 × RT buffer in the presence of random hexamers (final concentration 6 μM) and d(T)_23_VN (final concentration 5 μM). First-strand cDNA was synthesized using a ProtoScript II First Strand cDNA synthesis kit (NEB). Second-strand cDNA was synthesized using DNA polymerase I (NEB) and RNase H (NEB) with the presence of dUTP mix (dUTP, dATP, dCTP, dGTP; final concentration 1 mM). After end repair and dA tailing, the product was ligated with an Illumina Y-shaped adapter. The final product was treated with UDG (NEB) then PCR amplified with an index primer set. Library size and quality were checked using a Bioanalyzer 2100 (Agilent, Santa Clara, CA, United States). Four lanes of 150-bp paired-end sequencing were performed using a NovaSeq 6000 sequencer (Illumina, San Diego, CA, United States).

### Gene Expression, Differentially Expressed Genes, and Gene Ontology Analysis

FastQC was performed to check the quality of the reads, and seqtk^1^ was used to remove the reads that failed to achieve the quality score threshold (30). RNA-Seq reads were then aligned to the adaptor, tRNA, and ribosomal RNA reference sequences using Bowtie2 (v.2.3.4.1; doi: 10.1038/nmeth.1923) with default parameters. The remaining filtered reads were then mapped to the honey bee genome (GCA_000002195.1, Amel_4.5 assembly^2^) using HISAT2 (version 2.1.0) (Kim et al., 2015) with parameters [–max-intronlen 50000 -p 6 –dta-cufflinks]. Analysis of the differentially expressed genes (DEGs) was performed based on the number of mapped raw reads and identified using Bioconductor DEseq2 (v.1.12.4; Bioconductor 3.7) with default parameter and Wald test (Love et al., 2014). The DEGs with FDR (false discovery rate) < 0.05 and fold change ≥ 2 (log_2_ fold change ≥ 1 or ≤−1) were considered differentially expressed. The functional analysis and GO clustering of remaining DEGs were performed using the online gene functional classification tool, DAVID^3^ (Huang et al., 2009a; Huang et al., 2009b). Three tables were generated for each subset of DEGs, including “functional annotation clustering,” “functional annotation chart,” and “functional annotation table.”

## Results

### Gene Expression Estimation and Differentially Expressed Gene Identification

To monitor the effect of sublethal doses of imidacloprid on honey bee gene expression, we collected larvae and adults of worker bee at five different ages. The sampling time was determined based on the relative probability of task performance as described in Seeley (1982). For each age, a total of three biological replicates were collected, with five bees per replicate to a final 15 bees per age per treatment. Honey bees were collected from two beehives and then randomly assigned for each replicate; thus, the results represented in this report may eliminate the colony effects. Four lanes of 150 bp Illumina paired-end sequencing were generated; read yields per sample are shown in Supplementary Table 1A. Read counts for each gene, as well as FPKM levels at various development stages, are listed in Supplementary Tables 1B–F, 2A–E, respectively. Imidacloprid was supplied in a feeding solution using 0.1% DMSO in ddH_2_O as a solvent; control bees were fed with 0.1% DMSO solution without insecticide.

**FIGURE 1:**
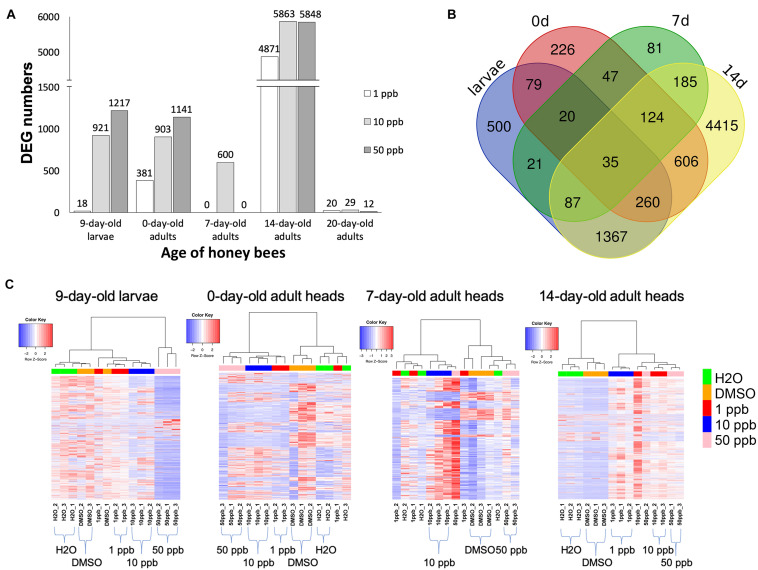
Numbers of differentially expressed genes at various developmental stages of worker bees. **(A)** Numbers of differentially expressed genes (DEGs) in 9-day-old larvae, as well as in 0-, 7-, 14-, and 20-day-old adults of honey bees exposed to 1-, 10-, or 50-ppb imidacloprid treatment for 4 days during the larval stage. Original numbers of DEGs are shown on top of each column. X-axis: the developmental stages of honey bees; Y-axis: the number of differentially expressed genes. **(B)** Venn diagram of shared DEGs of 9-day-old larvae, 0-, 7-, and 14-day-old adults among all concentrations of imidacloprid. The numbers of shared DEGs are shown in the overlapped region. Larvae: 9-day-old larvae; 0d: 0-day-old adults; 7d: 7-day-old adults; 14d: 14-day-old adults. **(C)** Cluster analysis of DEG expression patterns of 9-day-old honey bee larvae, as well as 0-, 7-, and 14-day-old adults. Read counts were used for cluster analysis. Complete normalization was applied, and the clustering was performed based on Pearson correlation coefficients. The expression trend of each replicate is shown. Controls (H_2_O and DMSO) and different concentrations of imidacloprid treatments (1, 10, and 50 ppb) are labeled in the bottom panel and marked with different colors in the panel of the dendrogram. Red: expression levels higher than average; blue: expression levels lower than average; white: expression levels close to average.

For each age of worker bee, we compared the gene expression levels between bees fed with imidacloprid and the 0.1% DMSO control to examine the DEGs to understand the impact of imidacloprid on honey bee gene expression at various ages. For each age of bee, three comparisons against the solvent control (bees fed with 1 μl of 0.1% DMSO) were performed: (i) 1 μl of 1 ppb imidacloprid solution; (ii) 1 μl of 10 ppb imidacloprid solution; and (iii) 1 μl of 50 ppb imidacloprid solution (Supplementary Figure 1B). DEGs with FDR values < 5% were considered differentially expressed and selected for further analysis. The DEGs were then filtered based on their log_2_ fold change. DEGs with a log_2_ fold change < 1 or >−1 were discarded, while genes with a log_2_ fold change ≥ 1 or ≤−1 were considered twofold differentially expressed. DEGs with a log_2_ fold change ≤−1 were defined as “down-regulated,” and DEGs with a log_2_ fold change ≥ 1 were defined as “up-regulated” (Table 1 and Figure 1A). The list of DEGs is shown in Supplementary Tables 3A,B. The detailed analysis procedure is described in section “Materials and Methods.”

**TABLE 1:** DEG numbers in different stages of honey bee fed with different concentrations of imidacloprid.

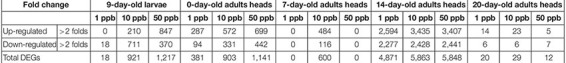

A cluster analysis was performed using read counts to visualize patterns of DEGs at various developmental stages (Figure 1C). Complete normalization was applied, and the clustering was performed based on Pearson correlation coefficients. For 9-day-old larvae, honey bees fed with 50 ppb imidacloprid showed distinct expression patterns and were assigned as the outgroup, while patterns of DEGs, between 1 and 10 ppb imidacloprid, and between H_2_O and DMSO, were similar, with only slight differences between one replicate of the DMSO solvent control and one replicate of the 1-ppb treatment compared to the other replicates. For 0-day-old adults, a clearer grouping between controls (DMSO and H_2_O) and imidacloprid treatments (1, 10, and 50 ppb) was observed, although one replicate of the 1-ppb treatment had expression patterns closer to the H_2_O control rather than the other two 1-ppb replicates. For 7-day-old adults, the clustering was chaotic and correlations between controls and treatments could barely be identified. For 14-day-old adults, with vast numbers of DEGs identified at this stage, significantly distinct groups between controls and imidacloprid treatments were observed without ambiguous distributions. Cluster analysis for 20-day-old adults was not performed as less than 30 DEGs were identified.

After being exposed to a sublethal dose of imidacloprid, the gene expression of honey bee larvae and adults was significantly affected, and the effects were long-lasting (Table 1). The number of DEGs increased with the dosage of imidacloprid in 9-day-old larvae (18, 921, and 1,217 DEGs for 1, 10, and 50 ppb, respectively) and 0-day-old adults (381, 903, and 1,141 DEGs for 1, 10, and 50 ppb, respectively). For 7-day-old adults, 600 DEGs were identified in bees fed with 10 ppb imidacloprid, while no DEGs were identified in the other treatments (1 and 50 ppb imidacloprid). Surprisingly, more than 4,800 DEGs were observed in 14-day-old adults (4,871, 5,863, and 5,848 DEGs for 1, 10, and 50 ppb, respectively), and the number of DEGs seems to have increased slightly with the dosage of imidacloprid. For 20-day-old adults, fewer than 100 DEGs were identified for all treatments (Table 1) (20, 29, and 12 DEGs for 1, 10, and 50 ppb, respectively). For 9-day-old larvae and 0-day-old adults, there are fewer upregulated than downregulated genes. In contrast, for 7-, 14-, and 20-day-old adults, a greater number of DEGs were identified in the upregulated groups.

The numbers of unique and shared DEGs among different concentrations of imidacloprid treatment at every age of bee were examined to evaluate the effect of the dosage of imidacloprid (Supplementary Figure 2). DEGs were first divided based on the regulatory trend (i.e., upregulated or downregulated), and then the shared DEGs were examined. The numbers of shared DEGs among the different concentrations of imidacloprid treatments are shown in Supplementary Figures 2A–D. In 9-day-old larvae and 0- and 20-day-old adults, the percentage of shared DEGs was less than 30%; more DEGs were found to be uniquely identified in the different concentrations of imidacloprid treatments. In 14-day-old adults, the percentage of shared DEGs was higher than 50%. The shared DEGs in 9-day-old larvae and 0- to 14-day-old adults were then examined among all the imidacloprid treatments, disregarding the regulatory trend. Thirty-five genes were identified as shared among different aged bees and different concentrations of imidacloprid treatments, including gamma-aminobutyric acid receptor subunit beta (GB40975), DNA ligase 1-like (GB42264), cuticular protein (GB46297), RNA polymerase II elongation factor EII (GB48521), insulin-like growth factor 2 mRNA-binding protein 1 (GB52056), nicotinamide riboside kinase (Nrk1) (GB53410), trehalose transporter 1 (GB55302), and several other unknown-function-genes (Figure 1B and Table 2).

**TABLE 2:** Functions of the 35 shared DEGS identified in four developmental stages.

**ID**	**Gene name**	**GO term, biological process**	**KEGG pathway**
GB40010	Titin-like (LOC551319)		
GB40975	Gamma-aminobutyric acid receptor subunit beta (LOC406124)		ame04080:Neuroactive ligand-receptor interaction
GB42169	NA		
GB42264	DNA ligase 1-like (LOC550914)		
GB43053	NA		
GB44068	NA		
GB44519	Nitrogen permease regulator 3-like protein (LOC412531)		
GB44890	Regulator of non-sense transcripts 1 (LOC409839)	GO:0000184∼nuclear-transcribed mRNA catabolic process, non-sense-mediated decay	ame03013:RNA transport,ame03015:mRNA surveillance pathway
GB45459	TGF-beta receptor type-1 (LOC550930)		ame04068:FoxO signaling pathway,ame04144:Endocytosis,ame04350:TGF-beta signaling pathway
GB45497	NA		
GB45705	NA		
GB45822	Corepressor interacting with RBPJ 1 (LOC724875)		ame04330:Notch signaling pathway
GB46297	Cuticular protein 14 (CPR14)		
GB47318	NA		
GB47946	NA		
GB48241	Protein N-terminal glutamine amidohydrolase (LOC725501)		
GB48521	RNA polymerase II elongation factor Ell (LOC409928)	GO:0006368∼transcription elongation from RNA polymerase II promoter	
GB48840	NA		
GB50065	Uncharacterized LOC100576569 (LOC100576569)		
GB50158	60S ribosomal protein L4 (LOC408526)	GO:0006412∼translation	ame03010:Ribosome
GB50423	Uncharacterized LOC408807 (LOC408807)		
GB51158	Uncharacterized LOC726321 (LOC726321)		
GB51272	NA		
GB51760	NA		
GB52056	Insulin-like growth factor 2 mRNA-binding protein 1 (LOC410398)		
GB52316	Kinectin (LOC409414)		ame04141:Protein processing in endoplasmic reticulum
GB52427	Plastin-2 (LOC408694)		
GB52925	NA		
GB53115	Apidermin 1 (Apd-1)		
GB53410	Nicotinamide riboside kinase (Nrk1)	GO:0019359∼nicotinamide nucleotide biosynthetic process	ame00760:Nicotinate and nicotinamide metabolism,ame01100:Metabolic pathways,
GB54351	MAP kinase-interacting serine/threonine-protein kinase 1 (LOC412470)		
GB55302	Trehalose transporter 1 (Tret1)	GO:0015771∼trehalose transport	
GB55368	NA		
GB55416	NA		
GB55598	NA		

*NA, not applicable.*

### Gene Ontology Analysis of DEGs

To further understand the biological meaning of the DEGs, Gene Ontology (GO) analysis was performed to predict their functions. DEGs of every treatment were first divided into two groups, i.e., upregulated and downregulated genes, based on their expression trend. An overview of the results of the “functional annotation chart” among all the treatments is shown in Supplementary Tables 3C,D. A GO term that could be identified in at least three ages of bee was considered as a constantly affected function. In the upregulated genes, GO terms identified in at least three ages of bees were as follows: Coiled coil, ion transport, nucleotide binding, Nucleotide-binding (alpha-beta plait), Ribosome, Ribonucleoprotein, Ribosomal protein, structural constituent of ribosome, Transport, and translation. Among the downregulated genes, most of the GO terms can be only identified in two ages of worker bee (Supplementary Tables 3C,D).

In general, the numbers of functional terms were correlated with the numbers of DEGs. For 9-day-old larvae, the number of downregulated clusters (79 and 35 for 10 and 50 ppb, respectively) was higher than that of upregulated clusters (7 and 14 for 10 and 50 ppb, respectively). The opposite pattern (i.e., a higher number of upregulated clusters) was observed in 0-, 7-, 14-, and 20-day-old adults. The numbers of functional terms of upregulated DEGs for 0-, 7-, 14-, and 20-day-old adults were as follows: 0-day-old adults, 16, 34, and 45 for 1, 10, and 50; 7-day-old adults, 23 for 10 ppb; and 14-day-old adults, 52, 91, and 81 for 1, 10, and 50 (Supplementary Table 3C). The numbers of GO cluster numbers of downregulated DEGs were as follows: 0-day-old adults, 8, 8, and 26 for 1, 10, and 50; 7-day-old adults, 10 for 10 ppb; and 14-day-old adults, 16, 27, and 37 for 1, 10, and 50 (Supplementary Table 3D). For upregulated terms, most of them were identified in 9-day-old larvae or 0-, 7-, and 20-day-old adults can be found in 14-day-old adults, while few functional clusters were only identified in 9-day-old larvae or 0-day-old adults. Terms such as ATPase/dynein-related/AAA domain, Dynein heavy chain, Frag1/DRAM/Sfk1, integral component of membrane, and transferase activity/transferring hexosyl groups were uniquely found in 9-day-old larvae, while 3′,5′-cyclic-nucleotide phosphodiesterase activity, 3′,5′-cyclic nucleotide phosphodiesterase/catalytic domain, Biotin/lipoyl attachment, Cyclic nucleotide-binding domain, Developmental protein, DnaJ domain, Groucho/transducin-like enhancer, Neuroactive ligand-receptor interaction, Nuclear hormone receptor/ligand-binding/core, Potassium channel, voltage-dependent, EAG/ELK/ERG, Ras-association, Serine-threonine/tyrosine-protein kinase catalytic domain, Steroid hormone receptor, Synaptotagmin, voltage-gated potassium channel activity, Zinc finger, and nuclear hormone receptor-type were found only in 0-day-old adults (Supplementary Table 3C). Terms of downregulated genes were found to be more diverse among different ages of bee, suggesting that the negatively affected genes may vary or change among different ages or tasks of worker bees. Higher numbers of DEGs were identified in 14-day-old adults than in 9-day-old larvae, yet fewer GO terms were identified in 14-day-old adults than in 9-day-old larvae. Nine-day-old honey bee larvae are transforming from larval to pupal, to finally adult stage, which may be involved in massive transcriptome reprogramming and diverse functional pathways for the differentiation and development of adults’ organs. High numbers of GO terms may suggest that various functional pathways could be affect after exposure to sublethal dose of imidacloprid.

The complete GO analysis results generated using DAVID are shown in Supplementary Table 4 (9-day-old larvae, 1 ppb imidacloprid treatment), Supplementary Tables 5A–F (9-day-old larvae, 10 ppb imidacloprid treatment), Supplementary Tables 6A–E (9-day-old larvae, 50 ppb imidacloprid treatment), Supplementary Tables 7A–F (0-day-old adults, 1 ppb imidacloprid treatment), Supplementary Tables 8A–F (0-day-old adults, 10 ppb imidacloprid treatment), Supplementary Tables 9A–F (0-day-old adults, 50 ppb imidacloprid treatment), Supplementary Tables 10A–F (7-day-old adults, 10 ppb imidacloprid treatment), Supplementary Tables 11A–F (14-day-old adults, 1 ppb imidacloprid treatment), Supplementary Tables 12A–F (14-day-old adults, 10 ppb imidacloprid treatment), Supplementary Tables 13A–F (14-day-old adults, 50 ppb imidacloprid treatment), Supplementary Tables 14A–D (20-day-old adults, 1 ppb imidacloprid treatment), Supplementary Tables 15A–C (20-day-old adults, 10 ppb imidacloprid treatment), and Supplementary Tables 16A,B (20-day-old adults, 50 ppb imidacloprid treatment).

### Differential Expression of Genes Related to Foraging Regulators at 14-Day-Old Adults

To examine whether the increasing amount of DEGs at 14-day-old adults is correlated with the cue for the switch of development of the worker bees, the expression trends of the key transcription factors that regulate the behavioral maturation in honey bees (Bloch et al., 2009) were examined. Among the 12 foraging regulators, eight including RXRA/RXRB (1, 10, and 50 ppb), CREB1 (10 and 50 ppb), DFD (1 and 10 ppb), DRI (1 and 50 ppb), EGR1/EGR2 (1, 10, and 50 ppb), PAX6 (10 ppb), HES1 (1, 10, and 50 ppb), and BHE40/BHE41 (10 and 50 ppb) were found upregulated in 14-day-old adults after imidacloprid treatment at the larval stage (Table 3; Supplementary Table 17A for read count of TF-related genes).

**TABLE 3:** Differentially expressed genes related to transcriptional regulators of honey bee behavioral state at different developmental ages after imidacloprid treatment.

	**Human ortholog**	**Fly ortholog**	**Bee gene**	**9-day-old larvae**		**0-day-old adults**			**7-day-old adults**	**14-day-old adults**		
				**10 ppb**	**50 ppb**	**1 ppb**	**10 ppb**	**50 ppb**	**10 ppb**	**1 ppb**	**10 ppb**	**50 ppb**
Nursing regulators	USF1/USF2	usf	GB40634									
	CXXC1	cfp1	GB43820			Up		Up	Up			
	MYOD1	Nautilus	GB55306									
Foraging regulators	RXRA/RXRB	Ultraspiracle	GB42692							Up	Up	Up
	CREB1	Creb-B17A	GB46492								Up	Up
	C/EBP	slbo	GB44204									
	DFD	Deformed	GB51299		Up					Up	Up	
	HXA1	Labial	GB51303			Up	Up	Up				
	ATF3	atf3	GB53401	Down								
	DRI	Retained	GB55596	Down				Up		Up		Up
	NF-κB	Dorsal	GB42472									
	EGR1/EGR2	Stripe	GB50091							Up	Up	Up
	PAX6	Eyeless	GB50342								Up	
	HES1	Hairy	GB47799		Down					Up	Up	Up
	BHE40/BHE41	cwo	GB52039			Up	Up				Up	Up

*This table is modified from Khamis et al. (2015).*

*up, upregulated gene; down, downregulated gene.*

### High Transcriptome Similarity Between Imidacloprid-Treated 14-Day-Old Bees and 20-Day-Old Control Bees

**FIGURE 2:**
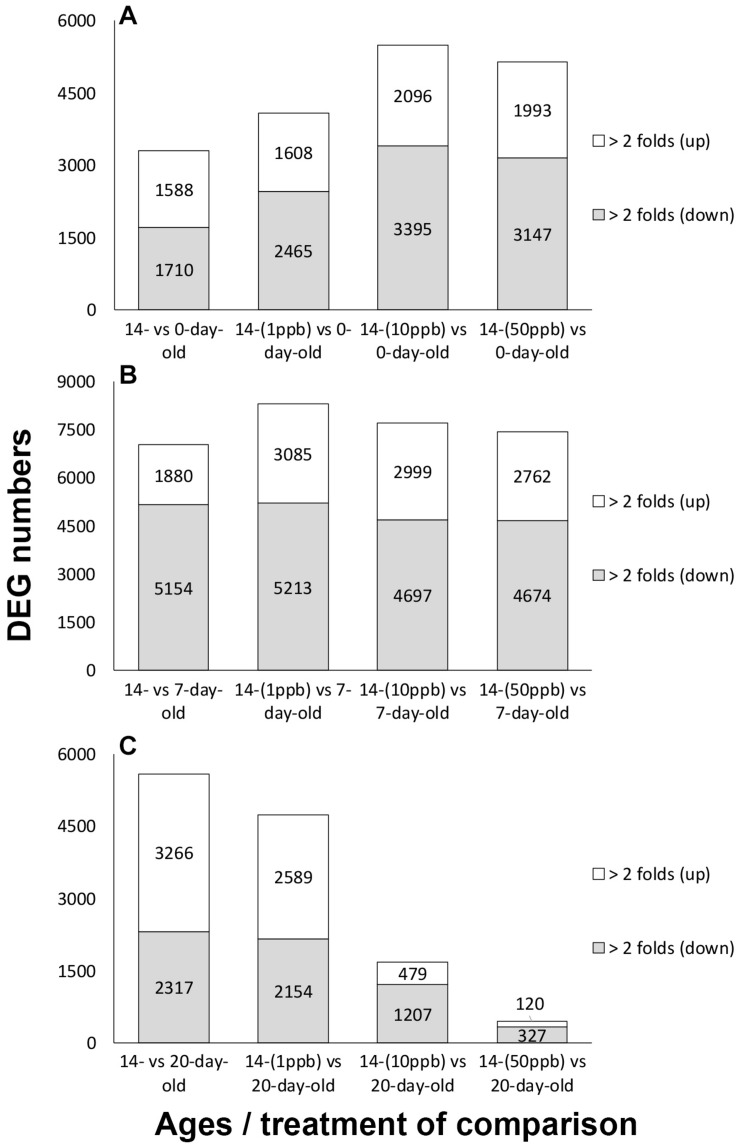
Numbers of differentially expressed genes (DEGs) identified from comparing 14-day-old imidacloprid treated bees with 0-, 7-, and 20-day-old control bees. **(A)** Fourteen-day-old imidacloprid treatments compared to 0-day-old adult control. **(B)** Fourteen-day-old imidacloprid treatments compared to 7-day-old adult control. **(C)** Fourteen-day-old imidacloprid treatments compared to 20-day-old adult control. DEGs were subdivided based on regulatory trends. Twofold (up): DEGs with a log_2_ fold change ≥ 1; twofold (down): DEGs with a log_2_ fold change ≤ –1. Numbers of DEGs are shown in each column.

The upregulation of foraging regulators in 14-day-old imidacloprid-treated adults suggests that the switch from nurse bee to forager was accelerated. To confirm this hypothesis, the transcriptome of 14-day-old bees treated with imidacloprid during larval stage was compared to that of 0-, 7-, and 20-day-old control bees (see Supplementary Figure 1C for a schematic diagram of comparison). A comparison between different ages of control bees was also performed (see Supplementary Figure 1D for a schematic diagram of comparison, Supplementary Figure 3 for results). Compared to 0- and 7-day-old controls, the total numbers of DEGs were 4,073 (14-day-old 1 ppb vs. 0-day-old control), 5,491 (14-day-old 10 ppb vs. 0-day-old control), 5,140 (14-day-old 50 ppb vs. 0-day-old control), 8,298 (14-day-old 1 ppb vs. 7-day-old control), 7,696 (14-day-old 10 ppb vs. 7-day-old control), and 7,436 (14-day-old 50 ppb vs. 7-day-old control) (Figures 2A,B). In the comparison of 14-day-old treatments to the 20-day-old control, the numbers of DEGs were negatively correlated with the concentration of imidacloprid. The numbers of DEGs identified from 14-day-old 1 ppb vs. 20-day-old control, 14-day-old 10 ppb vs. 20-day-old control, and 14-day-old 50 ppb vs. 20-day-old control were 4,743, 1,686, and 447, respectively (Figure 2C). The decreasing number of DEGs suggests the increase in transcriptome similarity, and the effect was dose-dependent. A cluster analysis of solvent controls and imidacloprid-treated workers in 9-day-old larvae and 0-, 7-, 14-, and 20-day-old adults was also performed to visualize expression patterns of whole transcriptome among different ages of bee. Fourteen-day-old workers with imidacloprid treatment during the larval stage were correlated with 20-day-old solvent control and treatments, suggesting the transcriptome similarity among them (Supplementary Figure 4).

## Discussion

### Fluctuation of DEG Numbers Among the Ages of Worker Bees

The number of DEGs decreased with the progress of worker bee development from 9-day-old larva to 7-day-old adult, peaked in 14-day-old adults, and then dramatically decreased to less than 30 in 20-day-old adults (Table 1 and Figure 1A). The different concentrations of imidacloprid seem to have induced different effects, as the DEGs were diverse (Supplementary Figures 2A–D). The high similarity of expression patterns between biological replicates in every treatment excludes the possibility of individual variance or artifacts, and distinct DEG patterns were observed between controls and imidacloprid treatments (Figure 1C and Supplementary Figure 5 for sample correlation). The fluctuating DEG numbers suggest that the global gene expression in the honey bee changes with the progress of their development as well as task performance. As nurse bees and foragers contain distinct transcriptomes (Khamis et al., 2015), it is highly possible that the gene expression profile would change with the different tasks performed by the nurse bees. The upregulation of the nursing regulator at 0-day-old adults suggests that those DEGs could be correlated with the development of the nursing behavior (Table 3), which should be performed while the hive bees become 3-day-old or older. A similar trend was also observed for the foraging regulators for 14-day-old adults (Table 3). The DEGs identified at 9-day-old larvae and 0- and 7-day-old adults may reflect not only the damages but also the early maturation induced by sublethal imidacloprid treatment. The decrease in DEGs from 9-day-old larvae to 7-day-old adults may suggest that the task performance of nurse bees is attributed by a small subset of genes. Further experiments are needed to validate the behavioral change of newly emerged workers and the task-associated genes of nurse bees.

### General Effects of Neonicotinoid Treatment Among Different Species of Insects

We compared our DEG results with previous studies (Forfert and Moritz, 2017; Shi et al., 2017a) and found that 329 DEGs were shared (Supplementary Table 18A). These genes are functionally related to lipid metabolism, pheromone/general odorant-binding protein, cytochrome P450, transmembrane transport, and ATP binding (Supplementary Tables 18B,C,D). Physiological experiments have confirmed that imidacloprid and clothianidin can impact energetic/nutrient homeostasis of honey bee (Cook, 2019). These genes could be toxification index genes expressed to inspect the existence of neonicotinoids in the honey bee. In bumble bee (*Bombus terrestris*) workers, exposure to field-realistic imidacloprid concentrations (10 ppb) can induce a total of 405 DEGs with functions related to energy reserve metabolism, synaptic transmission, apoptotic processes, xenobiotic transport, and complement activation, and including P450 genes (Manjon et al., 2018); exposure to a sublethal dose of clothianidin (6.47 ppb) induces the differential expression of 55 genes, including three putative cytochrome P450 genes (Bebane et al., 2019; Colgan et al., 2019). In *Bradysia odoriphaga*, exposure to the LC_50_ dose (4.08 mg/l) of imidacloprid for 24 h induces the differential expression of 674 unigenes with functions related to cytochrome P450, cell adhesion, axon guidance, and carbohydrate metabolic process (Chen et al., 2019). The effects of neonicotinoids on insect gene expression vary among different species of insects, but genes related to metabolism, detoxification (especially cytochrome P450), and neuron development/synaptic transmission seem to be commonly found. It is confirmed from molecular evidence that even at low concentrations, neonicotinoids can have an impact on the neuron development and detoxification of insects.

### Forager-Like Transcriptome Induced by Exposure to Sublethal Doses of Imidacloprid During Larval Stage

Honey bee division of labor correlates with the age of the adults, exposure to pheromones, ovary size, biogenic amines, and the proportion of members of the colony currently engaging in each task (Seeley, 1982; Huang and Robinson, 1992; Johnson, 2008, 2010; Bloch et al., 2009; Sagili et al., 2011; Wang et al., 2012; Reim and Scheiner, 2014). From a physiological perspective, a significant increase in the titers of JH coordinates the transformation from nurse bee to forager (Jaycox, 1967; Jaycox et al., 1974; Robinson, 1987; Fahrbach and Robinson, 1996; Bloch et al., 2009). From a molecular perspective, the switch from nurse bees to foragers is modulated by the differential expression of transcription factors and alternative transcription start sites, resulting in transcriptome changes followed by organized behavioral states (Whitfield et al., 2003; Khamis et al., 2015). In our study, it may not be easy to infer the trend of the JH titer based on the function of the identified DEGs, as the GO term related to JH synthesis/metabolite was not identified. Despite the expression of JH-related genes, the upregulation of the foraging regulators and the low transcriptomic difference between imidacloprid-treated 14-day-old adults and 20-day-old control adults suggest that the precocious worker bee may leave the beehive. Colin et al. (2019) examined the effect of a sublethal dose of imidacloprid by providing the beehive with a sucrose solution containing 5 ppb of the insecticide for 3 weeks and then monitored the foraging behavior of newly engaged foragers; foragers exposed to the insecticide started the orientation flight at an age 1.38 days younger than the control (Colin et al., 2019). Although the treatment methodology was different from the present study, it supports the relationship between the insecticide and precocious behavior. The occurrence of precocious foraging is believed to be triggered by a high mortality of foragers (Beshers and Fewell, 2001; Leoncini et al., 2004; Perry et al., 2015; Ushitani et al., 2016). The young foragers are found to be less efficient and have poor spatial memory and shorter lifespan (Chang et al., 2015; Perry et al., 2015; Ushitani et al., 2016) and cannot sustain the foraging task for long. This accelerated behavioral switch in our study is unlikely to be induced by the low number of foragers, as the experimental individuals were released into a healthy colony with task-performing foragers and food storage. We cannot exclude, however, that other unknown factors, such as pathogens or parasites, could have affected the task transition. Further validation and experiments are required to confirm if this phenomenon is a general effect, as two local colonies were used in this report.

### Differential Expression of Nicotinic Acetylcholine Receptor Genes

Imidacloprid acts as an agonist of the insect neuronal nicotinic acetylcholine receptors and would consequently affect the expression of nicotinic acetylcholine receptor genes (nAChRs) (Déglise et al., 2002; Eiri and Nieh, 2016). nAChRs mediate insect synaptic neurotransmission as well as many cognitive processes, including vision and olfactory learning (Breer and Sattelle, 1987). In the GO cluster analysis of DEGs, Nicotinic acetylcholine-gated receptor, transmembrane domain, was identified (Supplementary Tables 12A, 13A), although the numbers of nAChRs-related DEGs were related low [five out of 3,435 (10 ppb) and 3,407 (50 ppb)] and resulted in a high *p*-value. We examined the expression of nAChRs-related genes and found five of them that are differentially expressed in 14-day-old treated adults. These genes include nAChRa6 (GB43416; 1, 10, and 50 ppb), nAChRa8 (GB40923; 10 and 50 ppb), nAChRa9 (GB53427; 1 ppb), nAChRb1 (GB53055; 50 ppb), and nAChRa9 (GB53427; 10 and 50 ppb), which were upregulated, and nAChRb2 (GB53428, 10 and 50 ppb) which was downregulated (Table 4; Supplementary Table 17B for read counts). Among 11 nicotinic acetylcholine receptors subunit genes (Jones et al., 2006), four genes, including nAChRa2, nAChRa7, nAChRa8, and nAChRb1, are expressed in the Kenyon cells, the ordered neuropils in mushroom bodies that are crucial for olfactory learning and memory (Hammer and Menzel, 1995). Most of the differentially expressed nAChRs were found in 14-day-old treated adults regardless of the dose of imidacloprid. This might be correlated with the development of foraging behavior in adults over 10 days of age (Khamis et al., 2015). Food reward-induced olfactory stimuli are required to perform food collection and foraging behavior (Menzel, 1999; Dupuis et al., 2011; Giurfa and Sandoz, 2012). It is also possible for this differential expression to be associated with the development of precocious foraging behavior in the 14-day-old treated adults, as previously described due to the transcriptome similarity between these adults and the 20-day-old control. In the 35 shared DEGs among the four ages, we also found nicotinamide riboside kinase (Nrk1) (GB53410). This gene is induced in eukaryotes by the presence of nerve damage to protect damaged neurons from degradation (Belenky et al., 2007). Differential expression of Nrk1 among all ages of treated bees suggests that the neurons were damaged after the sublethal imidacloprid treatment. These results support previous findings that the exposure to sublethal doses of imidacloprid during the larval stage can induce neuron damage and consequently impair honey bee adults’ olfaction and related learning behavior (Medrzycki et al., 2003; Forfert and Moritz, 2017).

**TABLE 4:** Differentially expressed nAChRs family subunit genes in 14-day-old adults with 1-, 10-, and 50-ppb imidacloprid treatments.

**Bee stages**	**Gene ID**	**Imidacloprid treatments**	**Expression trend**	**Function**	**GO terms**	
14-day-old adults	GB43416	1, 10, 50 ppb	Up-regulated	Nicotinic acetylcholine receptor alpha6 subunit (nAChRa6)	GO:0016021	Integral component of membrane
					GO:0030054	Cell junction
					GO:0045211	Postsynaptic membrane
	GB40923	10, 50 ppb	Up-regulated	Nicotinic acetylcholine receptor alpha8 subunit (nAChRa8)	GO:0016021	Integral component of membrane
					GO:0030054	Cell junction
					GO:0045211	Postsynaptic membrane
	GB53427	1, 10, 50 ppb	Up-regulated	Nicotinic acetylcholine receptor alpha9 subunit (nAChRa9)	GO:0016021	Integral component of membrane
	GB53428	10, 50 ppb	Down-regulated	Nicotinic acetylcholine receptor beta2 subunit (nAChRb2)	GO:0016021	Integral component of membrane
	GB53055	50 ppb	Up-regulated	Nicotinic acetylcholine receptor beta1 subunit (nAChRb1)	GO:0016021	Integral component of membrane
					GO:0030054	Cell junction
					GO:0045211	Postsynaptic membrane

## Data Availability Statement

Honey bee RNA-seq raw reads are available at https://www.ncbi.nlm.nih.gov/sra/PRJNA521949.

## Author Contributions

E-CY and Y-RC designed the experiments. Y-RC, CT, P-SH, and T-HW performed the imidacloprid treatment and honey bee sampling. Y-RC prepared the RNA-Seq library. DT performed the data analysis. Y-RC, DT, E-CY, and SZ wrote the manuscript. All authors contributed to the article and approved the submitted version.

## Conflict of Interest

The authors declare that the research was conducted in the absence of any commercial or financial relationships that could be construed as a potential conflict of interest.

## Footnotes

1 https://github.com/lh3/seqtk

2 https://metazoa.ensembl.org/Apis_mellifera/Info/Index

3 https://david.ncifcrf.gov/gene2gene.jsp
