# Supplementary material for: Missing Nurse Bees—Early Transcriptomic Switch From Nurse Bee to Forager Induced by Sublethal Imidacloprid
Source: Front Genet. 2021 Jun 17;12:665927. doi: 10.3389/fgene.2021.665927 (PMC8248817; doi:10.3389/fgene.2021.665927)
Supplement: Supplementary file 2 [file Data_Sheet_2.docx]

**a**


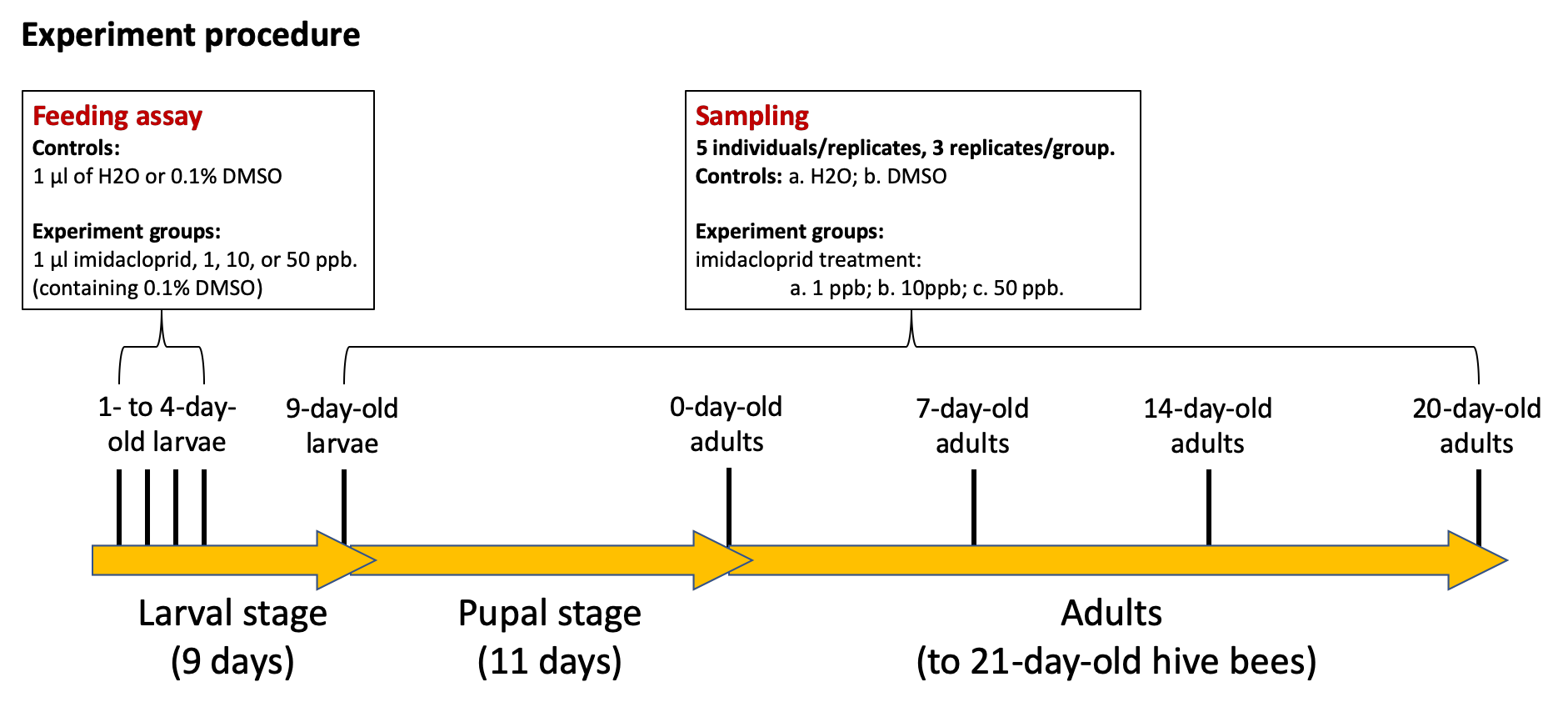


**b**


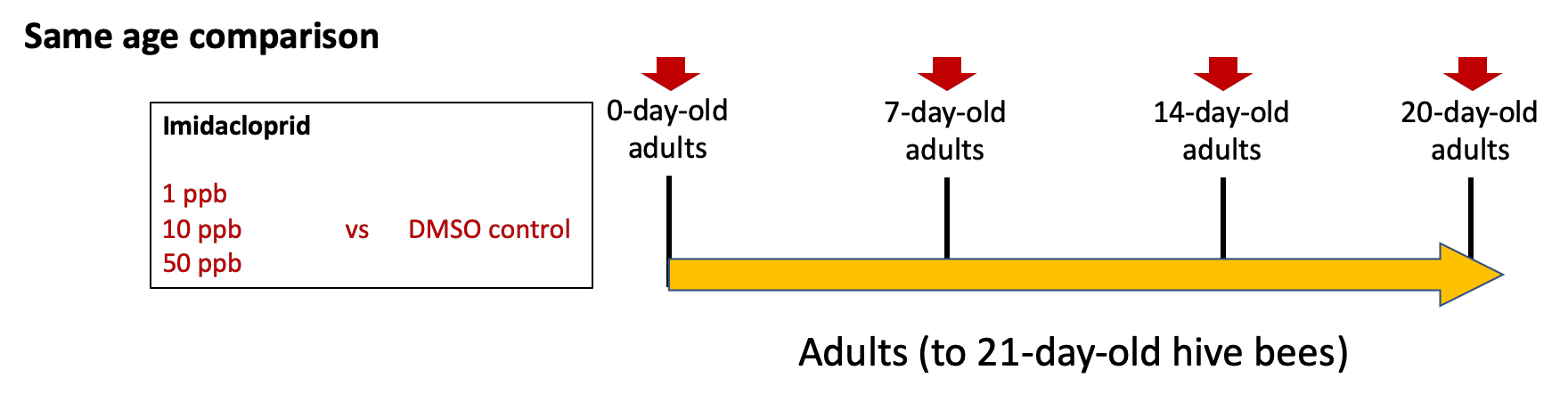


**c**


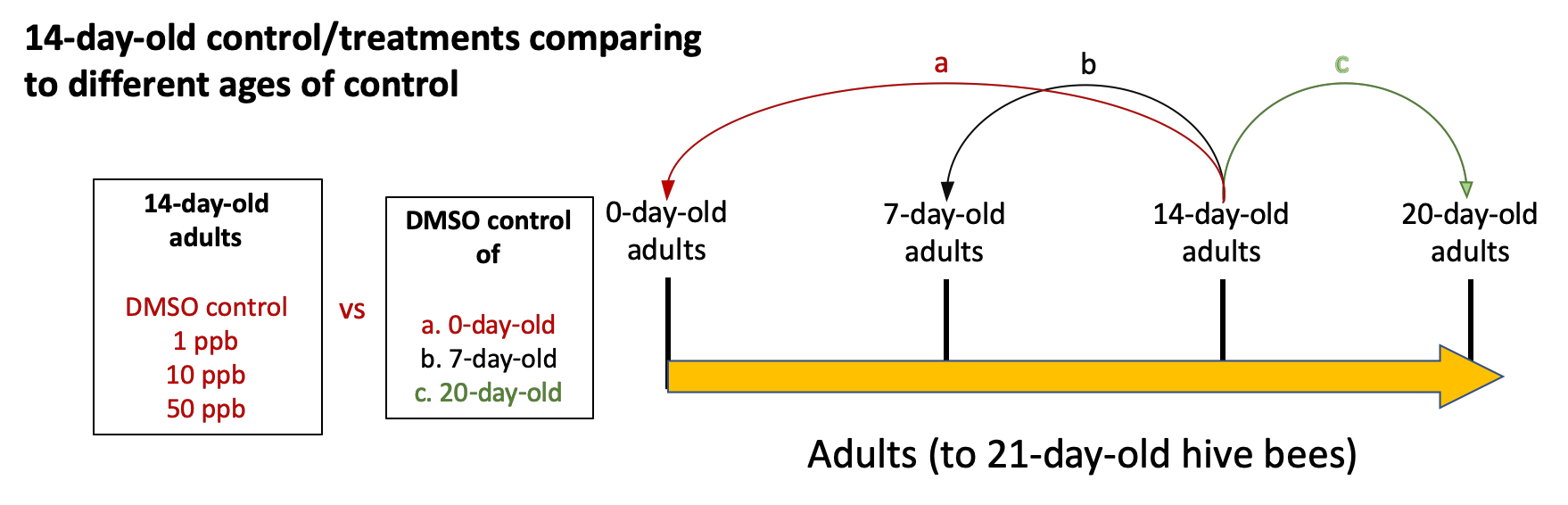


**d**


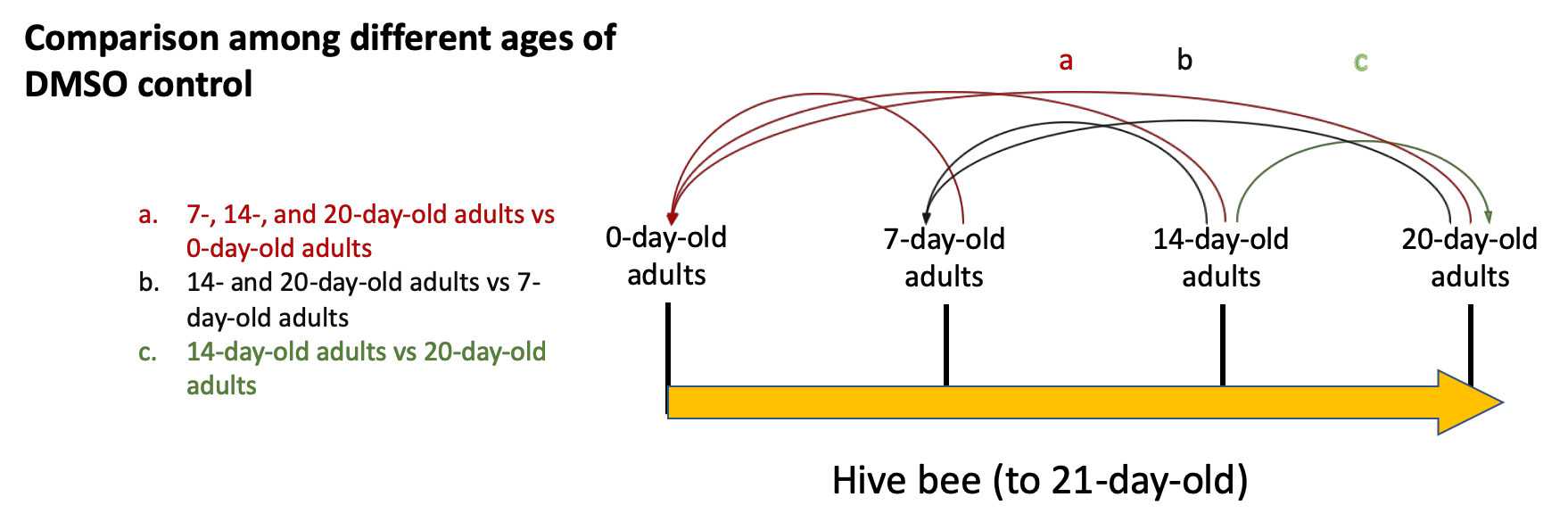


**Supplementary Fig. 1** **Schematic diagram of experiment procedure (a) and all the comparisons (b-d) in this study.** **a. Experiment procedure.** Imidacloprid treatment was performed during the larval stage. One microliter of H_2_O, 0.1% DMSO, 1, 10, or 50 ppb imidacloprid solution (containing 0.1% DMSO) was applied into the cell of 1-day-old larvae. This feeding procedure was performed daily for four days, from 1- to 4-day-old larvae, to mimic the number of days honey bee larvae consume bee bread. Sampling was performed on 9-day-old larvae, and 0- (newly emerged), 7-, 14-, and 20-day-old adults. The 0-, 7-, 14-, and 20-day-old adults; **b. Same age comparison**. Treatments were comparing to the same age control; **c. 14-day-old control/treatment comparing to different ages of control.** Gene expression profiles of DMSO control and 1ppb, 10ppb, and 50 ppb imidacloprid treated 14-day-old adults were comparing with that of DMSO control of 0-, 7-, and 20-day-old adults; **d. Comparison among different ages of DMSO control.** Comparing the gene expression profile of DMSO control among 0-, 7-, 14-, and 20-day-old adults. Arrow: indicating the comparison direction. Red line: comparing to DMSO control of 0-day-old adults; black line: comparing to DMSO control of 7-day-old adults; green line: comparing to DMSO control of 20-day-old adults.


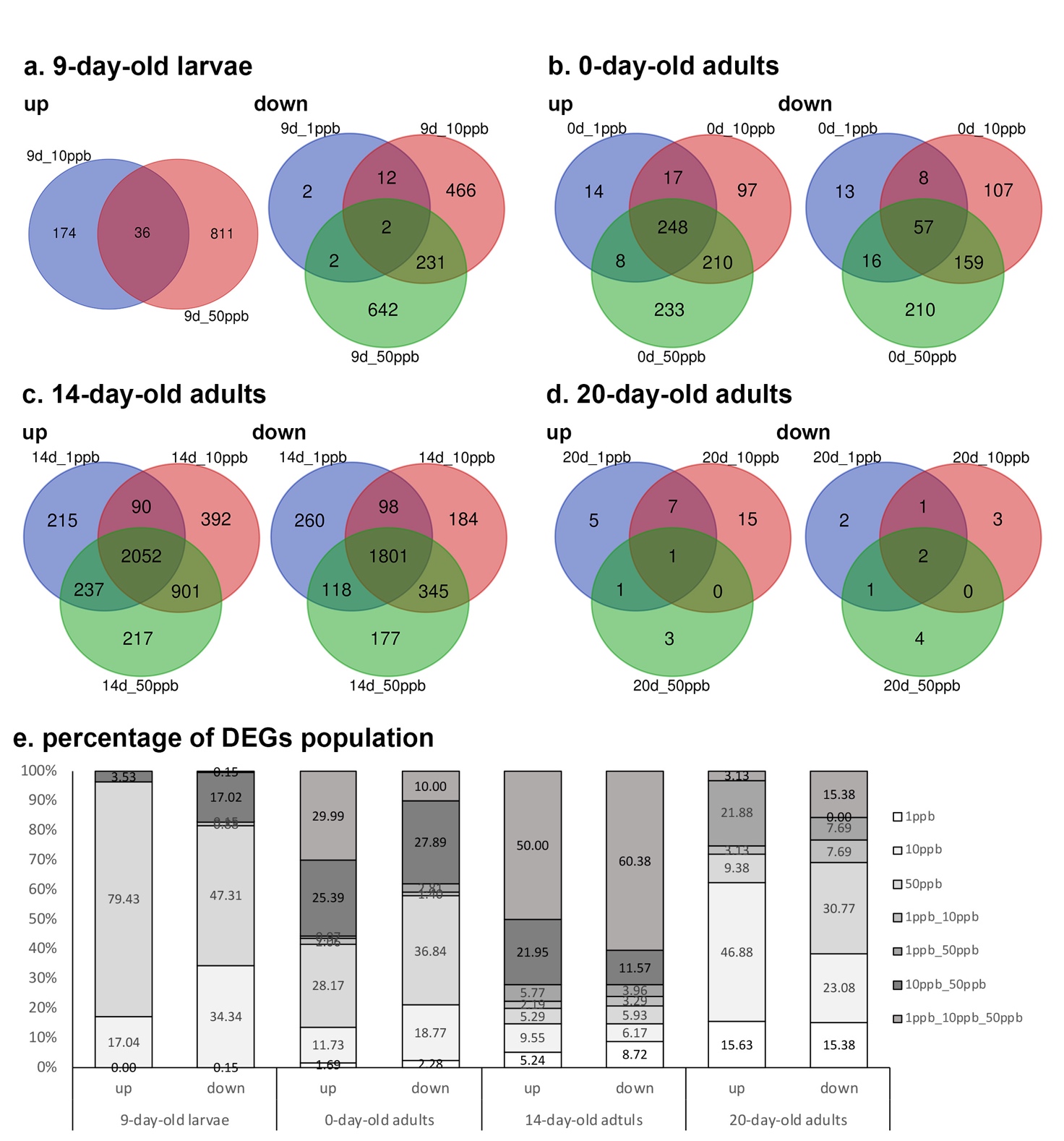


**Supplementary Fig. 2 Venn diagrams of shared DEGs numbers and the percentage of (a-d) shared DEGs population.** The shared DEGs numbers were shown as a. 9-day-old bee larvae, b. 0-day-old bee adults, c. 14-day-old bee adults, and d. 20-day-old bee adults with 1 ppb, 10pp, and 50 ppb imidacloprid treatment during larvae stages. The shared DEGs percentage (%) were shown as e. 1ppb: DEGs only identified in 1 ppb group. 10ppb: DEGs only identified in 10 ppb group. 50ppb: DEGs only identified in 50 ppb group. 1ppb_10ppb: DEGs shared between 1 ppb and 10 ppb groups. 1ppb_50ppb: DEGs shared between 1 ppb and 50 ppb groups. 10ppb_50ppb: DEGs shared between 10 ppb and 50 ppb groups. 1ppb_10ppb_50ppb: DEGs shared between 1 ppb, 10 ppb, and 50 ppb groups.


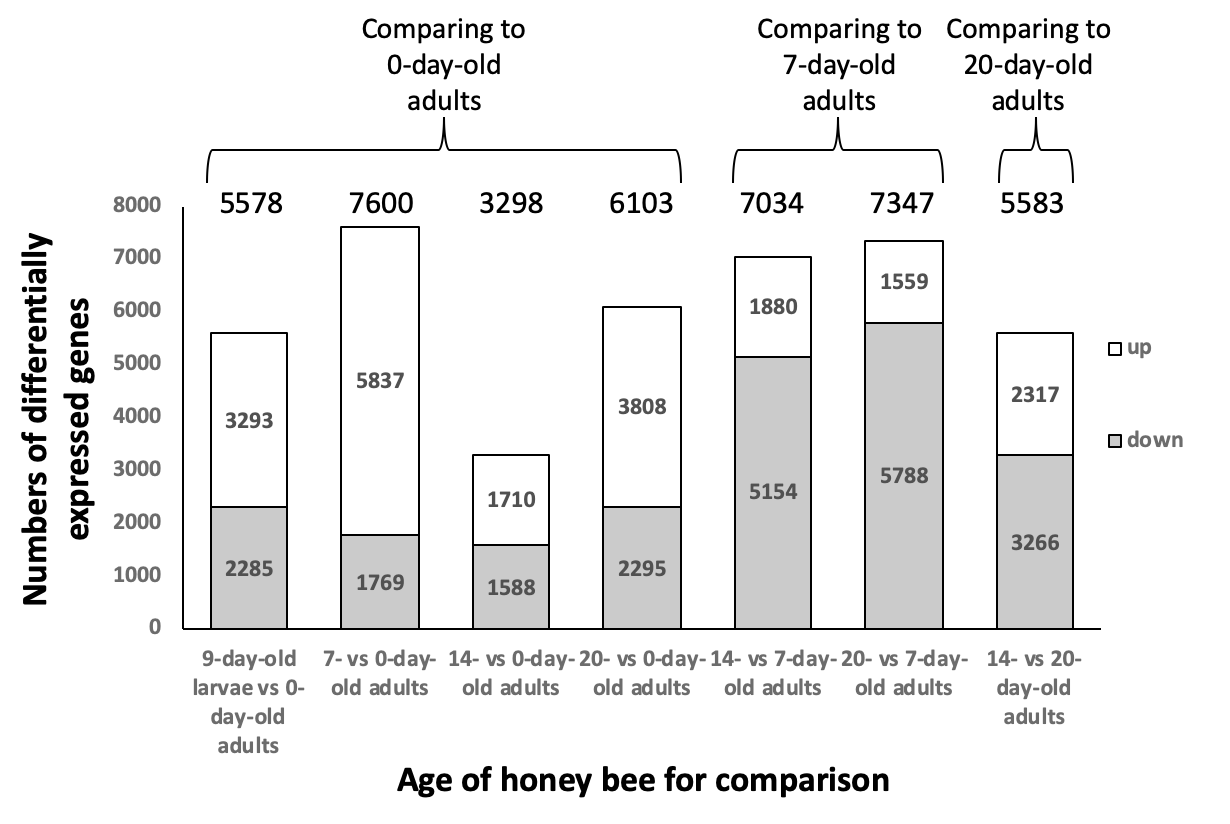


**Supplementary Fig. 3 Numbers of differentially expressed gene (DEG) identified from comparing different ages of control honey bee.** Schematic diagram for the comparison is shown in Supplementary Fig. 1d. Different ages of honey bee including 9-day-old larvae, 0-day-old adults, 7-day-old adults, 14-day-old adults, and 20-day-old adults were collected. Analysis of 9-day-old larvae vs 0-day-old adults, 7- vs 0-day-old, 14- vs 0-day-old, and 20- vs 0-day-old are using 0-day-old adults as control/standard to identify the DEGs; 14- vs 7-day-old and 20- vs 7-day-old are using 7-day-old adults as control/standard for analysis; 14-vs 20-day-old is using 20-day-old adults as control/standard for analysis. DEGs were subdivided based on the expression trend. DEGs were subdivided based on the regulatory trends. 2-folds (up): DEGs with log2foldchange ≥ 1; 2-folds (down): DEGs with log2foldchange ≤ -1. Numbers of DEGs were shown in each column, and the total numbers of DEG were labeled on the top panel.


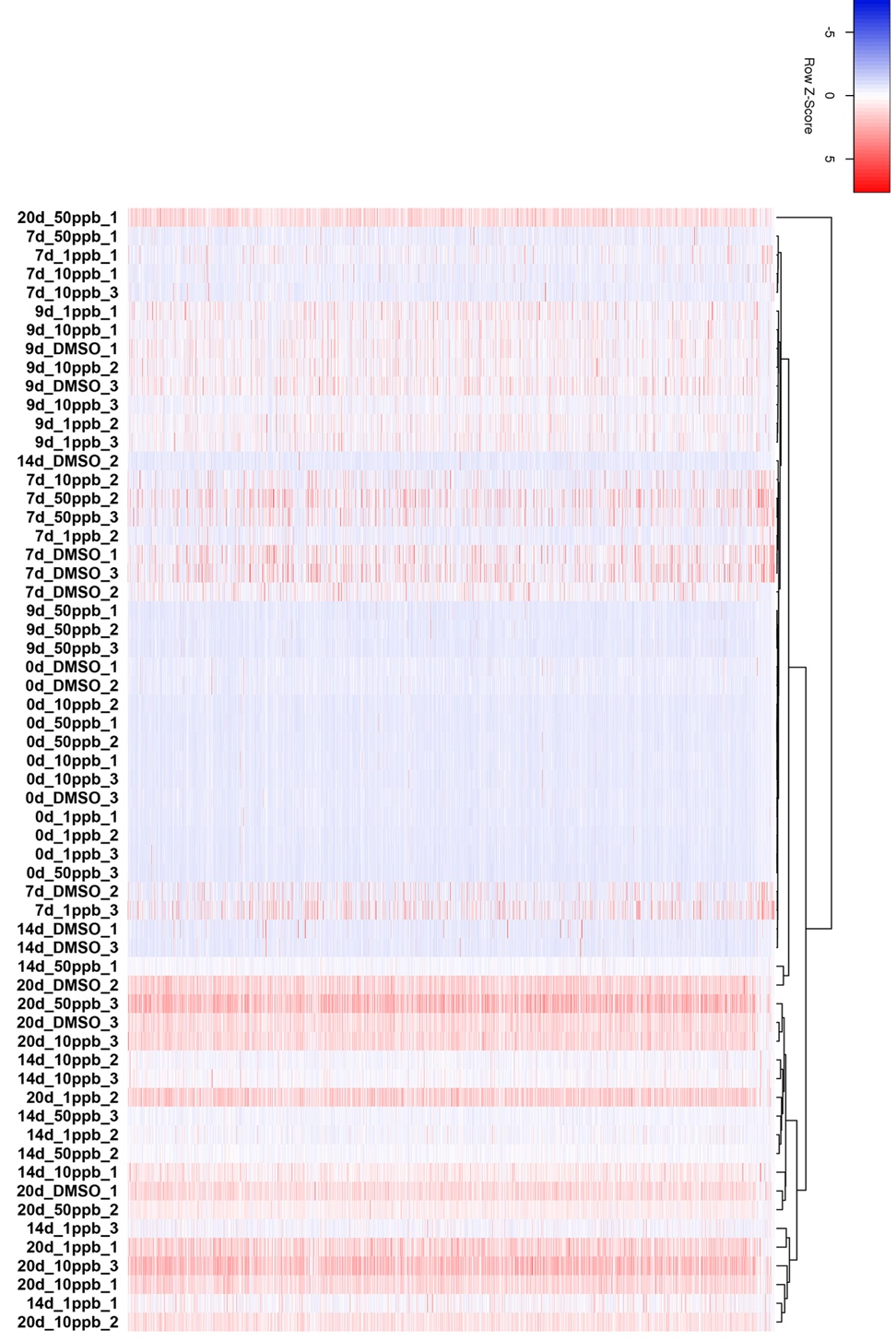


**Supplementary Fig. 4** Cluster analysis of whole transcriptomes of solvent control and 1, 10, and 50 ppb imidacloprid treated 9-day-old larvae as well as 0-, 7-, 14- and 20-day-old adults. Read counts were used for cluster analysis. Complete normalization was applied, and the clustering was performed based on Pearson correlation coefficients. The expression trend of each replicate is shown. Solvent control (0.1% DMSO), different concentrations of imidacloprid treatments, and the age of bee are labeled in the bottom panel. Red: expression levels higher than average; blue: expression levels lower than average; white: expression levels close to average. 9d_DMSO_1-3: 9-day-old larvae with solvent treatment, replicates 1-3; 9d_1ppb_1-3: 9-day-old larvae with 1ppb imidacloprid treatment, replicates 1-3; 9d_10ppb_1-3: 9-day-old larvae with 10ppb imidacloprid treatment, replicates 1-3; 9d_10ppb_1-3: 9-day-old larvae with 50ppb imidacloprid treatment, replicates 1-3; 0d_DMSO_1-3: 0-day-old adults with solvent treatment during larval stage, replicates 1-3; 0d_1ppb_1-3: 0-day-old adults with 1ppb imidacloprid treatment during larval stage, replicates 1-3; 0d_10ppb_1-3: 0-day-old adults with 10ppb imidacloprid treatment during larval stage, replicates 1-3; 0d_50ppb_1-3: 0-day-old adults with 50ppb imidacloprid treatment during larval stage, replicates 1-3; 7d_DMSO_1-3: 7-day-old adults with solvent treatment during larval stage, replicates 1-3; 7d_1ppb_1-3: 7-day-old adults with 1ppb imidacloprid treatment during larval stage, replicates 1-3; 7d_10ppb_1-3: 7-day-old adults with 10ppb imidacloprid treatment during larval stage, replicates 1-3; 7d_50ppb_1-3: 7-day-old adults with 50ppb imidacloprid treatment during larval stage, replicates 1-3; 14d_DMSO_1-3: 14-day-old adults with solvent treatment during larval stage, replicates 1-3; 14d_1ppb_1-3: 14-day-old adults with 1ppb imidacloprid treatment during larval stage, replicates 1-3; 14d_10ppb_1-3: 14-day-old adults with 10ppb imidacloprid treatment during larval stage, replicates 1-3; 14d_50ppb_1-3: 14-day-old adults with 50ppb imidacloprid treatment during larval stage, replicates 1-3; 20d_DMSO_1-3: 20-day-old adults with solvent treatment during larval stage, replicates 1-3; 20d_1ppb_1-3: 20-day-old adults with 1ppb imidacloprid treatment during larval stage, replicates 1-3; 20d_10ppb_1-3: 20-day-old adults with 10ppb imidacloprid treatment during larval stage, replicates 1-3; 20d_50ppb_1-3: 20-day-old adults with 50ppb imidacloprid treatment during larval stage, replicates 1-3.


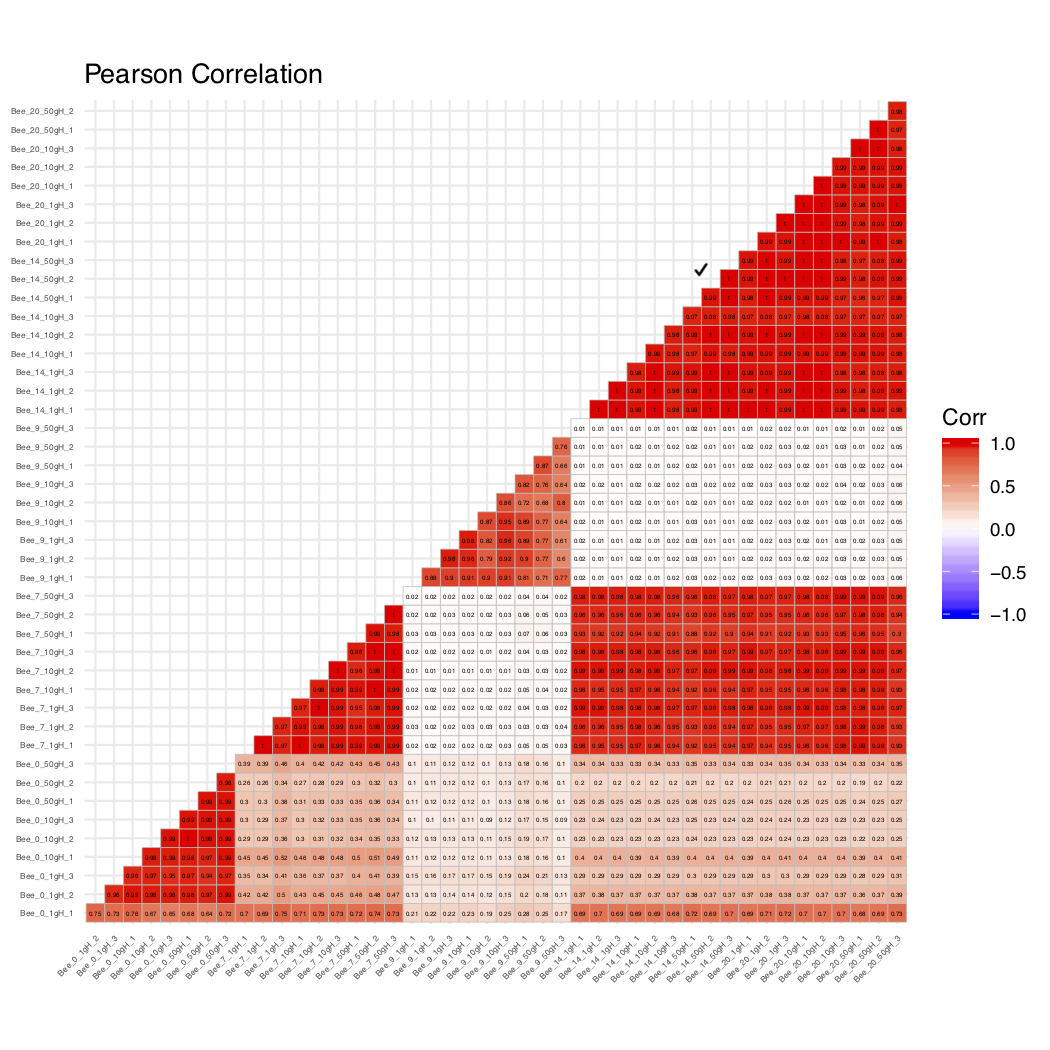


**Supplementary Fig. 5 Correlation heatmap of honey bee RNA-seq samples generated using ggcorrplot.** 9-day-old larvae with 1 ppt imidacloprid treatment: Bee_9_1gH_1, Bee_9_1gH_2, Bee_9_1gH_3; 9-day-old larvae with 10 ppt imidacloprid treatment: Bee_9_10gH_1, Bee_9_10gH_2, Bee_9_10gH_3; 9-day-old larvae with 50 ppt imidacloprid treatment: Bee_9_50gH_1, Bee_9_50gH_2, Bee_9_50gH_3.0-day-old adults with 1 ppt imidacloprid treatment: Bee_0_1gH_1, Bee_0_1gH_2, Bee_0_1gH_3; 0-day-old adults with 10 ppt imidacloprid treatment: Bee_0_10gH_1, Bee_0_10gH_2, Bee_0_10gH_3; 0-day-old adults with 50 ppt imidacloprid treatment: Bee_0_50gH_1, Bee_0_50gH_2, Bee_0_50gH_3.7-day-old adults with 1 ppt imidacloprid treatment: Bee_7_1gH_1, Bee_7_1gH_2, Bee_7_1gH_3; 7-day-old adults with 10 ppt imidacloprid treatment: Bee_7_10gH_1, Bee_7_10gH_2, Bee_7_10gH_3; 7-day-old adults with 50 ppt imidacloprid treatment: Bee_7_50gH_1, Bee_7_50gH_2, Bee_7_50gH_3.14-day-old adults with 1 ppt imidacloprid treatment: Bee_14_1gH_1, Bee_14_1gH_2, Bee_14_1gH_3; 14-day-old adults with 10 ppt imidacloprid treatment: Bee_14_10gH_1, Bee_14_10gH_2, Bee_14_10gH_3; 14-day-old adults with 50 ppt imidacloprid treatment: Bee_14_50gH_1, Bee_14_50gH_2, Bee_14_50gH_3.20-day-old adults with 1 ppt imidacloprid treatment: Bee_20_1gH_1, Bee_20_1gH_2, Bee_20_1gH_3; 20-day-old adults with 10 ppt imidacloprid treatment: Bee_20_10gH_1, Bee_20_10gH_2, Bee_20_10gH_3; 20-day-old adults with 50 ppt imidacloprid treatment: Bee_20_50gH_1, Bee_20_50gH_2, Bee_20_50gH_3.
